# Determinants of Acute Malnutrition among Children Aged 6–59 Months in Public Health Facilities of Pastoralist Community, Afar Region, Northeast Ethiopia: A Case Control Study

**DOI:** 10.1155/2017/7265972

**Published:** 2017-09-13

**Authors:** Anwar Seid, Berhanu Seyoum, Firehiwot Mesfin

**Affiliations:** ^1^College of Medical and Health Sciences, Samara University, Samara, Ethiopia; ^2^College of Health and Medical Sciences, Haramaya University, Harar, Ethiopia

## Abstract

**Background:**

In low income countries, acute malnutrition continues to be the most important risk factor for illnesses and deaths. The aim of this study was to assess the determinants of acute malnutrition among children aged 6–59 months.

**Methods:**

A facility based unmatched case control study was employed on 420 (140 cases and 280 controls) children aged 6–59 months with their caregivers between January 20 and February 20, 2014. Data was analyzed using SPSS version 20.0. A *P* value < 0.05 was considered statistically significant.

**Results:**

Children aged 12–23 months [AOR = 10.51, 95% CI = 4.93, 22.34], rural residence [AOR = 2.42, 95% CI = 1.22, 4.79], illiterate father [AOR = 2.47, 95% CI = 1.32, 4.61], Monthly income of less than 1000 birr [AOR = 3.98, 95% CI 2.05, 7.69], and food served together with family [AOR = 2.18, 95% CI = 1.10, 4.30] were associated with acute malnutrition.

**Conclusion:**

Rural residence, illiterate father, monthly income of less than 1000 birr, and food served together with family are statistically associated with acute malnutrition. Improving practices of parents on appropriate child feeding and creating awareness related to key risk factors of acute malnutrition should be further strengthened.

## 1. Background

Acute malnutrition is an extremely severe disorder. It is associated with high rates of morbidity and mortality. Globally, 52 million children under five years of age were wasted and more than 29 million children under five years suffered from severe wasting [1]. Stunting, severe wasting, intrauterine growth restriction, and low birth weight together were responsible for about 2.1 million deaths and 91.0 million disability adjusted life years at global level [2]. Moreover, severe acute malnutrition is the commonest reason for paediatric hospital admission. This is because an estimated 2 million children under five were admitted for treatment of severe acute malnutrition globally [1].

The burden of acute malnutrition is not evenly distributed around the world. Approximately 1-2% of the under-five population in low income countries are affected by severe acute malnutrition, which may be responsible for about 1 million child deaths every year [3]. In sub-Saharan Africa, nearly 1 in 10 children under the age of five were wasted. More than 80 percent of the 2 million children treated for acute malnutrition were found in sub-Saharan Africa [1].

Ethiopia is one of the countries with high burden of acute malnutrition. The country is among the top ten most affected countries by wasting (10% of under-five children wasted, of which 3% were severely wasted in the year 2011) [1, 4]. In Afar Region, prevalence of wasting among under-five children was 19.5%. Out of these, 6.2% of them were severely wasted [4]. At the national level, the trend in nutritional status of the children shows significant changes regarding stunting and underweight but the prevalence of wasting is not as such showing improvement.

According to the 2005 EDHS report, the prevalence of wasting in Afar Region was 9.9%, of which 2.9% were severely wasted [5]. However, there are limited data on acute malnutrition in the study setting. Therefore, the present study was conducted to determine factors associated with acute malnutrition among 6–59 months old children in Dubti District, Afar Region, and Northeast Ethiopia.

## 2. Methods

### 2.1. Study Design, Period and Setting

A health facility based unmatched case control study design was conducted between January 20, 2014, to February 20, 2014. The study was conducted in Dubti District, Afar Region, Northeast Ethiopia. Dubti District is one of the Districts in Awsi Zone of Afar Region, which is found in the Lower Awash Valley about 574 km North East of Addis Ababa and 13 km far from the regional capital city, Samara. According to the 2007 Central Statistical Agency report, the total population of children aged 0–5 years was 8787. In Dubti District, there are 12 health facilities (one referral hospital, one health center, and 10 health posts).

### 2.2. Study Population and Sampling Procedure

The study population were children of age between 6 and 59 months, visiting the nutrition units and other health services in the health facilities found in Dubti District. This study had two groups of participants; the first group includes children of age between 6 and 59 months with mid upper arm circumference of less than 12 cm in children whose length is >65 cm and the second comprises children of age between 6 and 59 months with mid upper arm circumference ≥ 12 cm.

The sample size was calculated by using Epi info version 3.3.2 statistical software. We consider the following assumptions: the number of children greater than three in the household as the exposure variable which is 40.2% among those who are acutely malnourished (cases) and 25.5% among those who are not malnourished children (control) with the odds ratio of 1.96 [6], 95% level of confidence, power of 80%, and 1 : 2 case to control ratio. The sample sizes became 127 cases and 254 controls. By adding 10% nonresponse rate for each group, the final sample size was 140 cases and 280 controls (in total 420).

Both health center and referral hospital found in the district were included and 140 children who were acutely malnourished and 280 well-nourished but visited/admitted to the hospitals for other healthcare issues were selected. Children aged 6–59 months with acute malnutrition were allocated to the health facilities depending on the average previous month's acutely malnourished children flow to the health facilities.

From previous months on average 80 children with cases of acute malnutrition were reported and during the two months there were 160 children with cases of acute malnutrition at Dubti Referral Hospital. Depending on this, 62 cases and 124 controls were allocated to Dubti Referral Hospital and systematic random sampling technique was used to select every 2nd child from 160 children.

In Detibahri Health Center from previous months on average 60 children with cases of acute malnutrition were reported in one month and during two months there were 120 children with cases of acute malnutrition and systematic random sampling technique was used to select every 2nd child. Depending on this 47 cases and 94 controls were allocated to Detibahri Health Center.

Similarly in Dubti Health Center monthly flow of acute malnutrition was 40 and during the two-month period 80 children with cases of acute malnutrition were reported. Based on this, 31 cases and 62 controls were allocated and finally the cases were selected every 2nd child. In order to increase the power of the study once a case was found and his or her caregiver interviewed, two controls meeting the criteria were selected and their caregivers interviewed from the same health facilities. The procedure was continued in the three selected health facilities throughout the data collection period until the required sample size was achieved.

### 2.3. Data Collection

Data were collected from all eligible study participants using a structured questionnaire which was adapted from Ethiopian National Nutrition Survey Questionnaire [7]. The questionnaire covered a range of topics including socioeconomic and sociodemographic factors, child caring practices, maternal characteristics, and environmental health conditions. MUAC was measured using the armband/tape (easily portable measurement device) to identify the cases and the controls. The questionnaire was translated to Afaraf (local language) and finally back to English language by language experts to check for its consistency. Then the questionnaire was pretested in another health facility on six cases and 12 controls. For data collection, six trained nurses and two supervisors were involved. Continuous supervision was made by the investigators during the data collection period.

### 2.4. Data Management and Analysis

The data were entered in to Epi-data version 3.2 and analysed using SPSS version 20.0. Logistic regression analysis was performed to identify factors leading to acute malnutrition. Odds ratio with 95% confidence interval was calculated to measure level of association between independent and dependent variables.

### 2.5. Ethical Considerations

The study was reviewed and approved by Haramaya University, College of Health and Medical Sciences, Institutional Health Research Ethical Review Committee (IHRERC). Official support letter was also written by Haramaya University to Dubti District. Written consent was obtained from each parents/caregivers of the children before initiation of data collection. To maintain confidentiality of information collected from each study participant, names and other identifiers were not used in the questionnaire.

## 3. Results

### 3.1. Sociodemographic and Socioeconomic Profile

In this study, a total of 420 study participants (140 cases and 280 controls) were involved. The mean age of the cases and controls was 15 months (SD ± 8) and 28 months (SD ± 16), respectively. Of the cases and the controls, 68 (48.6) and 156 (57.7) were males, respectively. Regarding the residence of the children, 92 (65.7) of the cases and 70 (25) of controls were from rural. The majority, 128 (91.4) of the cases and 246 (87.9) of the controls, were Muslims. Concerning the ethnicity of the participants, 106 (75.7) of the cases and 157 (56.1) of the controls were Afar (Table 1).

### 3.2. Child Feeding Practices

All of the cases and controls were ever breastfed. Breastfeeding was initiated after one hour of birth in 78 (55.7) of the cases and 145 (51.8) of the controls. Prelacteal feeding was given more frequently in 94 (67.1) cases than in the controls, 160 (57.1). Colostrum feeding was practiced in 93 (66.4) of the cases and 175 (62.5) of the controls. Seventy-five (53.6) and 184 (65.7) mothers of the cases and the controls were reported that they started additional food to their child at six months and beyond, respectively (Table 2).

### 3.3. Characteristics of Mothers of the Cases and the Controls Children

Almost half, 71 (50.7) of the mothers of the cases, and 75 (26.8) of the controls had no antenatal care follow-up. Concerning taking of extra food during pregnancy and lactation, 85 (60.7) of cases and 144 (51.4) of controls did not take additional food (Table 3).

### 3.4. Environmental Health and Sanitation Characteristics of the Cases and the Controls

More in the controls used water from protected source (244 (87.1)) than the cases, 82 (58.6). Availability of latrines was more in the controls, 251 (89.6), than the cases 80 (57.1) (Table 4).

### 3.5. Factors Associated with Acute Malnutrition

In multivariate analysis age of the child, current residence of the child, marital status, paternal education, monthly income, ways of preparing complementary food, ever receive vitamin A supplementation, birth order of the child, and water fetching time were statistically associated with acute malnutrition (Table 5).

## 4. Discussion

In this study, acute malnutrition was associated with children aged between 6 and 23 months, rural residence, married and in union family, paternal illiteracy, household monthly income of less than 1000 birr, ways of food serving and preparation, vitamin A supplementation, birth order, and mothers who are engaged in fetching water from walking a distance of less than or equal to 30 minutes (to and from).

Age of the child was one of the determinants of acute malnutrition. Children aged between 6 and 11 months and 12 and 23 months were 4 and 10 times more likely to be acutely malnourished than those aged 24–59 months, respectively. This result is consistent with study conducted in Nigeria which shows children of age between 12 and 23 months were more likely to be wasted than older ones [8]. Similarly according to the 2011 Ethiopian Demographic Health Survey Report, acute malnutrition was the highest in children aged 9–11 months (19 per cent) and the lowest in children aged 36–47 months (6 percent) [4]. Another study conducted in Gumbrit also showed children's age was significantly associated with acute malnutrition (*p* < 0.001) [9]. This is probably due to inappropriate feeding practice at earlier age and then later after two years the improvement seen may be due to the fact that the child can demand and take adult type of food when he/she needs.

The current study revealed that place of residence was strongly associated with acute malnutrition and children living in rural kebele were more likely to be acutely malnourished than children living in urban kebele (AOR = 2.42, 95% CI = (1.22, 4.798)). This finding was in agreement with study conducted in Nghệ An, Vietnam, which revealed that children in rural areas were 7.1 times more likely to be wasted when compared with children in urban areas [10]. Moreover, the higher prevalence of wasting was found among children living in rural areas of Bangladesh [11]. Also in Ethiopia according to Ethiopian Demographic Health Survey Report 10 percent of children in rural areas are wasted, compared with 6 percent in urban areas [4].

In this study being married and living in union with husband were found to be associated with not having acutely malnourished child. The chance of the child to be acutely malnourished was 65% less likely among children whose mothers were married and in union as compared to children whose mothers were single/widowed/divorced. This might be because the presence of both parents in the family was reflective of the better resources and childcare practices in households.

In the current study, children having an illiterate father were 2 times more likely to be acutely malnourished than those whose fathers were able to read and write. Equally the findings of this study agree with a case control study conducted in Gonder which revealed that children having an illiterate father were 2 times more likely to have acute malnutrition than those having literate fathers [6]. Probably educated fathers are better aware about the nutrition requirements of their children and they usually provide improved healthcare as a result of their awareness.

Household monthly income had showed significant association with acute malnutrition. The chance of the child to be acutely malnourished increases when households' monthly income was found to be below 1000 birr per month. This shows that household monthly income was inversely related to acute malnutrition. This finding is in line with other studies conducted both in country and abroad [6, 9, 11, 12]. The reason for malnutrition being more rampant among those household with lower monthly income may be due to their lower purchasing capacity for food and unavailability of hygienic and healthy living environment among them.

Ways of food serving and preparation were associated with acute malnutrition and those children who were served food with family and for whom food is prepared with the family were 2 times more likely to be acutely malnourished than those who served separately and for whom food is prepared separately. Similarly, a survey in North Wollo showed that the most important risk factors for acute malnutrition were child feeding practices specifically; the practice in which the child ate from the same plate with the rest of the household members [13]. This might be because children need more time to eat the right amount of food and they cannot compete when served with family.

In the current study children who receive vitamin A supplementation for the last 12 months were 48% less likely to have acute malnutrition as compared to children who were not supplemented with vitamin A. This might be because vitamin A supplementation reduced the prevalence of diarrhea by boosting the immunity which in turn decreases the risks of acute malnutrition.

Birth order of the child was significantly associated with acute malnutrition. As the birth order of the child in the family decreases the chance of the child to be acutely malnourished decreases. Children of birth orders one and two to three were 94% and 90% less likely to be acutely malnourished than those of birth order six and above, respectively. This finding is in agreement with other studies conducted in country and abroad [10, 11, 13]. This might be because when the birth order of the child in the household increases the care given to the children decreases because the mother becomes old and unable to provide appropriate care for those late comers. Moreover, when the family number increases it causes strain on family resources.

In this study, children whose mothers were engaged in fetching water from walking a distance of less than or equal to 30 minutes (to and from) were 87% less likely to be acutely malnourished than those whose mothers were fetching water by walking more than or equal to 60 minutes. This finding is in line with study conducted in North Wollo [13]. This might be because in the absence of suitable child minders extended separation of children from their primary caregivers for purpose of fetching water from long distance may be detrimental to child feeding. Also the heavy physical burden coupled with the lack of time for rest places heavy nutritional stress on women and affects the care given to the child. In addition, where water is not readily available and accessible, food hygiene is often inadequate. This again increases the risk of acute malnutrition.

## 5. Conclusion and Recommendation

This study confirmed those children aged between 6 and 23 months, rural residence, married and in union, paternal illiteracy, and household monthly income of less than 1000 birr are the socioeconomic and sociodemographic risk factors for acute malnutrition. Regarding child characteristics and feeding practice, ways of food serving and preparation, vitamin A supplementation, and birth order are strongly associated with acute malnutrition. Mothers who are engaged in fetching water from walking a distance of less than or equal to 30 minutes (to and from) are less likely to be acutely malnourished than long distance fetchers.

Thus, an organized effort should be made at all levels to strengthen interventions aimed at improving the practice of parents on ways of feeding for the child and safety net program implemented in the district to focus on the poorest segment of societies. Similarly the Regional Health Office should develop appropriate and locally feasible awareness creation campaigns to promote birth spacing and risk factors of acute malnutrition.

## Figures and Tables

**Table 1:** Sociodemographic and socioeconomic profile of cases and controls in Dubti District, Afar Regional State, Northeast Ethiopia, January 2014 (cases: *N* = 140 and controls: *N* = 280).

Variable	Profile	Cases, *N* (%)	Controls, *N* (%)
Child age (in months)	6–11	43 (30.7)	58 (20.7)
	12–23	64 (45.7)	51 (18.2)
	24–59	33 (23.6)	171 (61.1)
Sex of the child	Male	68 (48.6)	156 (55.7)
	Female	72 (51.4)	124 (44.3)
Current residence	Rural	92 (65.7)	70 (25)
	Urban	48 (34.3)	210 (75)
Religion	Muslim	128 (91.4)	246 (87.9)
	Orthodox	12 (8.6)	27 (9.6)
	Protestant	0 (0)	7 (2.5)
Ethnicity	Afar	106 (75.7)	157 (56.1)
	Amhara	24 (17.1)	100 (35.7)
	Oromo	5 (3.6)	15 (5.4)
	Tigre	5 (3.6)	8 (2.9)
Maternal education	Illiterate	104 (74.3)	151 (53.9)
	Read and write	36 (25.7)	129 (46.1)
Maternal marital status	Married	109 (77.9)	252 (90)
	Single/widowed/divorced	31 (22.1)	28 (10)
Paternal education	Illiterate	80 (57.1)	89 (31.8)
	Read and write	60 (42.9)	191 (68.2)
Maternal occupation	Unemployed	87 (62.2)	191 (68.2)
	Self-employed	16 (11.4)	21 (7.5)
	Employed	37 (26.4)	68 (24.3)
Family size	<5	67 (47.9)	212 (75.7)
	≥6	73 (52.1)	68 (24.3)
No of children in the HH	Less than or equal to three	62 (44.3)	218 (77.9)
	Greater than three	78 (55.7)	62 (22.1)
Livestock owned	Yes	91 (65)	132 (47.1)
	No	49 (35)	148 (52.9)
Monthly income (in birr)	<1000	70 (50)	56 (20)
	≥1000	70 (50)	224 (80)

**Table 2:** Child feeding practice of cases and controls from Dubti District, Afar Regional State, Northeast Ethiopia, January 2014 (cases: *N* = 140 and controls: *N* = 280).

Variable	Profile	Cases, *N* (%)	Controls, *N* (%)
BF initiation time	With in one hour	62 (44.3)	135 (48.2)
	After one hour	78 (55.7)	145 (51.8)
Colostrum feeding	Yes	93 (66.4)	175 (62.5)
	No	47 (33.6)	105 (37.5)
Prelacteal feed given	Yes	94 (67.1)	160 (57.1)
	No	46 (32.9)	120 (42.9)
EBF for 6 months	Yes	68 (48.6)	176 (62.9)
	No	72 (51.4)	104 (37.1)
Initiation of CF at 6 months	Yes	75 (53.6)	184 (65.7)
	No	65 (46.4)	96 (34.3)
Ways of preparing CF	Together with adult	54 (38.6)	61 (21.8)
	Separately	86 (61.4)	219 (78.2)
Frequency of CF	Once	23 (16.4)	35 (12.5)
	2-3 times	88 (62.9)	158 (56.4)
	4-5 times	21 (15)	58 (20.7)
	More than five	8 (5.7)	29 (10.4)
Hand washing	Yes	114 (81.4)	240 (85.7)
	No	26 (18.6)	40 (14.3)
Diarrhoea in the last two weeks	Yes	34 (24.3)	40 (14.3)
	No	106 (75.7)	240 (85.7)
Place to treat the child	Health facility	91 (65)	249 (88.9)
	Traditional healer	37 (26.4)	29 (10.4)
	Do nothing	12 (8.6)	2 (0.7)
Vaccination	Yes	114 (81.4)	255 (91.1)
	No	26 (18.6)	25 (8.9)
Vitamin A supplementation	Yes	51 (36.4)	205 (73.2)
	No	89 (63.6)	75 (26.8)
Birth order of the child	One	9 (6.4)	89 (31.8)
	2-3	57 (40.7)	146 (52.1)
	4-5	51 (36.4)	39 (13.9)
	≥6	23 (16.4)	6 (2.1)

BF = breast feeding; EBF = exclusive breast feeding; CF = complementary feeding.

**Table 3:** Characteristics of the mothers of cases and controls from Dubti District, Afar Regional State, Northeast Ethiopia, January 2014 (cases: *N* = 140 and controls: *N* = 280).

Variables	Profile	Cases, *N* (%)	Controls, *N* (%)
Age of the mother (in years)	16–25	24 (17.1)	78 (27.9)
	26–35	75 (53.6)	165 (58.9)
	36–45	41 (29.3)	37 (13.2)
Extra food taken	Yes	55 (39.3)	136 (48.6)
	No	85 (60.7)	144 (51.4)
Antenatal care follow-up	Yes	69 (49.3)	205 (73.2)
	No	71 (50.7)	75 (26.8)
Number of ANC visits	One	5 (7.2)	3 (1.5)
	Two	7 (10.1)	14 (6.8)
	Three	30 (43.5)	72 (35.1)
	Above three	27 (39.1)	116 (56.6)

**Table 4:** Environmental health and sanitation practice of cases and controls from Dubti District, Afar Regional State, Northeast Ethiopia, January 2014 (cases: *N* = 140 and controls: *N* = 280).

Variables	Profile	Cases, *N* (%)	Controls, *N* (%)
Source of water	Protected	82 (58.6)	244 (87.1)
	Unprotected	58 (41.4)	36 (12.9)
Water fetching time	≤30	99 (70.7)	268 (95.7)
	31–59	12 (8.6)	3 (1.1)
	≥60	29 (20.7)	9 (3.2)
Availability of latrine	Yes	80 (57.1)	251 (89.6)
	No	60 (42.9)	29 (10.4)
Type of latrine	Traditional pit latrine	77 (96.2)	225 (89.6)
	Ventilated pit latrine	3 (3.8)	26 (10.4)
Place to dispose child's excreta	Backyard	107 (76.4)	123 (43.9)
	Toilet	33 (23.6)	157 (56.1)
Solid waste disposal methods	Open field	83 (59.3)	89 (31.8)
	Dumping and burning	57 (40.7)	191 (68.2)

**Table 5:** Determinant factors of acute malnutrition among children aged between 6 and 59 months from Dubti District, Afar Regional State, Northeast Ethiopia, January 2014 (cases: *N* = 140 and controls: *N* = 280).

Variable	Cases, *N* (%)	Controls, *N* (%)	Crude OR (95% CI)	Adjusted OR (95% CI)
Child age (in months)				
6–11	43 (30.7)	58 (20.7)	3.84 (2.33, 6.61)	3.99 (1.77, 8.97)
12–23	64 (45.7)	51 (18.2)	6.50 (3.85, 0.97)	10.51 (4.93, 22.34)
24–59	33 (23.6)	171 (61.1)	1	1
Residence				
Rural	92 (65.7)	70 (25)	5.75 (3.69, 8.94)	2.42 (1.22, 4.79)
Urban	48 (34.3)	210 (75)	1	1
Maternal marital status				
Married	109 (77.9)	252 (90)	0.39 (0.22, 0.68)	0.36 (0.16, 0.82)
Single/widowed/divorced	31 (22.1)	28 (10)	1	1
Paternal education				
Illiterate	80 (57.1)	89 (31.8)	2.86 (1.88, 4.34)	2.46 (1.32, 4.61)
Read and write	60 (42.9)	191 (68.2)	1	1
HH monthly income				
<1000	70 (50)	56 (20)	4 (2.57, 6.22)	3.98 (2.05, 7.69)
≥1000	70 (50)	224 (80)	1	1
Ways of preparing CF				
Together with adult	54 (38.6)	61 (21.8)	2.25 (1.44, 3.51)	2.18 (1.11, 4.30)
Separately	86 (61.4)	219 (78.2)	1	1
Ever receive Vit. A				
Yes	51 (36.4)	205 (73.2)	0.21 (0.14, 0.32)	0.52 (0.29, 0.94)
No	89 (63.6)	75 (26.8)	1	1
Birth order of the child				
One	9 (6.4)	89 (31.8)	0.03 (0.01, 0.08)	0.059 (0.015, 0.23)
2-3	57 (40.7)	146 (52.1)	0.10 (0.04, 0.26)	0.09 (0.03, 0.32)
4-5	51 (36.4)	39 (13.9)	0.34 (0.13, 0.92)	0.38 (0.11, 1.32)
≥6	23 (16.4)	6 (2.1)	1	1
Water fetching time				
≤30	99 (70.7%)	268 (95.7%)	0.12 (0.05, 0.25)	0.128 (0.04, 0.37)
31–59	12 (8.6%)	3 (1.1%)	1.24 (0.29, 5.39)	0.625 (0.10, 3.82)
≥60	29 (20.7%)	9 (3.2%)	1	1

HH = household.
